# Modeling net energy requirements of 2 to 3-week-old Cherry Valley ducks

**DOI:** 10.5713/ajas.19.0561

**Published:** 2019-11-12

**Authors:** Ting Yang, Lexiao Yu, Min Wen, Hua Zhao, Xiaoling Chen, Guangmang Liu, Gang Tian, Jingyi Cai, Gang Jia

**Affiliations:** 1 Institute of Animal Nutrition, Sichuan Agricultural University, Chengdu, 611130, China; 2 College of Animal Science and Technology, Tibet Vocational Technical College, Lasa, 850000, China

**Keywords:** Duck, Net Energy Requirement, Factorial Method, Comparative Slaughter Method, Prediction Equation

## Abstract

**Objective:**

A total of three hundred unsexed ducks were utilized to estimate net energy requirements of maintenance (NE_m_) and weight gain (NE_g_) for 2 to 3-week-old Cherry Valley ducks and to establish a model equation to predict NE requirements using the factorial method.

**Methods:**

To determine the apparent metabolizable energy (AME) of the diet, fifty 7-day-old ducks at approximately equal body weights (BWs) were randomly assigned into five groups that were fed at different levels (*ad libitum*, 85%, 75%, 65%, and 55% of *ad libitum* intake), and the endogenous acid-insoluble ash as indigestible marker. The two hundred and fifty 7-day-old ducks were used for a comparative slaughter experiment. At the beginning of the experiment, ten ducks were sacrificed to determine the initial body composition and energy content. The remaining ducks were randomly assigned into five groups (same as metabolic experiment). Ducks of the *ad libitum* group were slaughtered at 14 and 21-day-old. At the end of the experiment, two ducks were selected from each replicate and slaughtered to determine the body composition and energy content.

**Results:**

The results of the metabolizable experiment showed AME values of 13.43 to 13.77 MJ/kg for ducks at different feed intakes. The results of the comparative slaughter experiment showed the NE_m_ value for 2 to 3-week-old Cherry Valley ducks was 549.54 kJ/kg of BW^0.75^/d, and the NE_g_ value was 10.41 kJ/g. The deposition efficiency values of fat (K_f_) and crude protein (K_p_) were 0.96 and 0.60, respectively, and the values of efficiency of energy utilization (K_g_) and maintenance efficiency (K_m_) were 0.75 and 0.88, respectively.

**Conclusion:**

The equation for the prediction of NE requirements for 2 to 3-week-old Cherry Valley ducks was the following: NE = 549.54 BW^0.75^+10.41 ΔW, where ΔW is the weight gain (g).

## INTRODUCTION

Accurate estimates of the nutritional requirements of animals and the nutritive value of feed ingredients are essential when formulating feed for different species of animals. Energy systems based on digestible energy (DE) value or metabolizable energy (ME) value are widely used in the poultry and pig industries. For broiler chickens, the relative efficiency of energy utilization for carbohydrate, fat and protein was determined to be 100%, 113%, and 78%, respectively [1]. The ME system undervalued the utilizable energy of fats and overestimated that of proteins in comparison with that of carbohydrates because the heat increment (HI) was not accounted for as energy loss [2]. The net energy (NE) system can accurately assess the energy requirements of animals and reflects the true energy value of feedstuffs that are used to feed animals, such as unconventional feedstuff [3,4]. As the energy requirements of humans continue to increase, and therefore, the adoption of the NE system to fully use unconventional feedstuff to alleviate this energy shortage is critical, such as food by-products. In the pig industry, a savings of 4.00 to 4.50 €/t of feed cost is possible, and the excretion of nitrogen is also reduced when the ME system is replaced by the NE system, without negative effects on production performance [5,6]. The nitrogen retained in diets with co-products is not affected when the imbalances in the amino acid concentrations or the post-absorptive energy metabolism of diets is taken into account [7]. In recent years, the ME or the DE systems have been substituted gradually by the NE system in pig husbandry of North America and Europe [8].

The NE requirements of animals can be determined using the factorial method that divides the NE requirements into maintenance (NE_m_) and production (NE_p_) requirements [9]. In general, maintenance requirements are independent of animal production level and nutritional composition of feed and are only related to the state of the animal [10]. The HI depends on environmental temperature, the amount of feed consumed, and also varies with the feeding time and duration of fasting, the most important factor of variation of HI is the diet composition [10–12]. The heat production (HP) of an animal can be determined using indirect calorimetry, and the fasting heat production (FHP) of an animal is commonly used to estimate the NE_m_ [13]. In previous studies, indirect calorimetry was used to determine the values of ingredients and NE requirements of ruminants, pigs, broilers, and breeder pullets [14,15]. However, Milgen and Noblet [16] raised objections to this method because the locomotor activity of animals is restricted, then, the collected data may not reflect the true HP of animals reared under normal conditions. The previous level of intake also affect the FHP because the fasting status influences the animal physiological state, which is associated with HP of animals [10,15,17]. Therefore, another method for the determination of the NE_m_ of animals is the regression method, as proposed by Lofgreen and Garrett [18]. The regression method mostly avoids the metabolic change caused by starvation, and the NE value is more accurate than that obtained with the calorimetric method. For poultry, the requirements of NE for growth, based on compounds that are deposited in the body, can be determined via the comparative slaughter method. Thus, we determined the NE requirements of ducks by adopting the regression and comparative slaughter methods.

The NE system has been widely adopted in the pig industry in some countries with developed animal husbandry, but for poultry, the adoption of the NE system is in the initial stage because information remains scarce and data on poultry NE implementation continue to be accumulated. Therefore, the objective of this study was to establish an equation to predict the NE requirements for 2 to 3-week-old Cherry Valley ducks by determining the NE requirements of maintenance (NE_m_) and weight gain (NE_g_) using the regression and comparative slaughter methods, respectively.

## MATERIALS AND METHODS

### Animal care

The experimental procedures for animal trials were conducted in accordance with the Chinese guidelines for animal welfare and approved by the Animal Health and Care Committee (IACUC) of Sichuan Agricultural University (SAU-14-158).

### Animal, experimental design and diets

A total of three hundred unsexed, 1-day-old Cherry Valley ducks (male and female) were reared in cage pens (1 m×1 m) at the farm of the Animal Nutrition Institute of Sichuan Agricultural University. The ducks (initial weight 55.37±0.26 g) were acclimated to the environment for 7 days. Ducks were fed with pellet form, and free access to feed and water throughout the experiment. The diet was based on corn-soybean meal and formulated to meet or slightly exceed NRC (1994) nutrient recommendations (Table 1). The temperature maintained at 32°C during the first week, and gradually decrease to 24°C at the end of the third week and then kept constant thereafter. All ducks were housed in an environmentally controlled facility, with continuous 24 h lighting.

In order to determine the apparent metabolizable energy (AME) of the experimental diet at different feeding level. A total of fifty 7-day-old ducks at approximately equal body weights (BWs, regardless of sex) were randomly assigned into five treatments with five replicates, each replicate containing two birds. The birds were provided diet at five levels of feed (*ad libitum* and 85%, 75%, 65%, and 55% *ad libitum* intake). The intake of feed restriction was confirmed by intake of ducks allowed *ad libitum* intake of previous day, and the experiment lasted eight days. The endogenous acid-insoluble ash (AIA) as an indigestible marker to measure the AME of experimental diets during this period.

A total of two hundred and fifty 7-day-old ducks were used for the comparative slaughter experiment. At the beginning of the experiment, ten ducks at approximately equal BWs were sacrificed to determine the initial body composition and energy content. The remaining ducks were randomly assigned into five groups (same as the metabolic experiment); the *ad libitum* group was replicated ten times, and the restriction groups were replicated five times, each replicate with eight ducks. At 14 days of age, ten ducks (BWs) were selected from five replicates of *ad libitum* group and sacrificed to determine the body composition. At 21 days of age, two ducks (BWs) were selected from each of the replicates were sacrificed to determine the final body composition.

### Sample collections and chemical analysis

For the metabolic experiment, excreta were collected three times per day for three days. The feathers, skin debris and spilled feed were carefully removed, the excreta were weighed, 10 mL of 10% hydrochloric acid and drops of toluene were added, and the excreta was stored at −20°C. At the end of collection, the collected excreta was pooled by replicating and dried in a forced-ventilation greenhouse for 72 h at 65°C. After milling of excreta, the samples were sent for analyses of dry matter, energy and AIA.

For the comparative slaughter experiment, ducks were fasted overnight by withdrawal of feed only. After weighing, ducks were euthanized by cervical dislocation, and the carcasses were frozen at −20°C with feathers and viscera. The frozen ducks were chopped into small pieces, and the pieces were passed through the grinder five times to prepare representative samples for analyses. The carcasses samples were weighed, homogenized and dried in a forced-ventilation oven for 96 h at 55°C. After 24 h, the carcasses samples were reweighed and ground to determine the body composition.

According to the procedure of AOAC (Association of Official Analytical Chemists) [19], the diet, excreta and carcass sample were dried at 105°C for 8 h to determine the dry matter. Total nitrogen of carcass sample was assayed using the Kjeldahl method, and ether extract of carcass sample was determined by extraction in anhydrous ether in a Soxhlet apparatus for 12 h. The gross energy of diet, excreta and carcass sample were measured in an automatic oxygen bomb calorimeter (PARR 6400; PARR Instruments CO., Moline, IL, USA). The AIA of diet and excreta were measured according to ISO 5985-2002.

### Data calculation

The BW gain was determined by calculating the difference in weight between the beginning and the end of the experiment. The amounts of feed and orts were weighed daily to calculate the feed intake. The data obtained from the metabolic experiment were used to calculate the AME of the diet using the endogenous indicator method.

The retained energy (RE) of body was determined by the difference between the initial and the final carcasses energy content (BE). The HP was defined as the difference between ME intake (MEI) and RE [20]. The NE_m_ was calculated form the antilog of the y-axis intercepts of the linear regression between the Log_10_HP and MEI [18]. The ME requirements for maintenance (ME_m_) were calculated from the regression equation between the RE and MEI, with the intercept on the x-axis representing the ME_m_ and the slope of regression between RE and MEI above maintenance representing the efficiency of ME utilization for retained energy (K_g_) [21]. The efficiency of ME utilization for maintenance (K_m_) was calculated as NE_m_ divided by ME_m_.

The NE_g_ was obtained from the linear regression equation between BE and BW, and the slope of the equation was the NEg. The ME requirements of weight gain (ME_g_) were calculated as NE_g_ divided by Kg. The total efficiency of energy deposition as protein (K_p_) and fat (K_f_) was determined by multiple linear regression of MEI and RE as protein and fat according to the model proposed by Boekholt et al [22]. The deposition protein was calculated as nitrogen of carcass multiplied by 6.25. The energy requirement of deposition as fat and protein are 39.22 kJ/g and 23.69 kJ/g, respectively [20]. The K_p_ and K_f_ are the efficiency of energy utilization of deposited protein and fat in this formula, respectively.

### Statistical analysis

Data are shown as the means±standard error, and were analyzed using the SPSS 19.0 (SPSS Inc., Chicago, IL, USA). Normal distribution and homogeneity of variance using were checked the Shapiro-Wilk and Levene’s tests, respectively. When data exhibited normally distributed and homogeneity, they were analysed using one-way analysis of variance and followed by the Duncan’s comparison. In addition, the linear regression equations about Log_10_HP, MEI, and RE, MEI were established by the general linear model procedure, and the R^2^ was used to compare these regressions. Statistical significance is defined as p<0.05

## RESULTS

### The performance and body composition of ducks

The results for duck performance at the different levels of intake are summarized in Table 2. The ADFI and ADG of ducks decreased and the feed conversion ratio (FCR) increased with decreasing feed intake, except between the *ad libitum* group and the 15% restriction group; therefore, the feed conversion increased when ducks were in a state of hunger. Table 3 shows the results of energy balances and body retained parameters. The BE and fat deposition declined as the supply of feed decreased. By contrast, protein deposition and carcasses moisture increased with the decrease in the level of intake. No significant difference was observed between the 15% and 25% restriction groups for carcasses moisture, and protein deposition was also not different between similar restriction groups (i.e., *ad libitum* and 15% restriction group and 35% and 45% restriction groups).

### Energy requirements for maintenance of ducks

In the experiment to determine the AME values, the apparent metabolizable rate of energy (AM) for the *ad libitum* group of ducks was 79.44%, which increased slightly to 81.39% (55% *ad libitum* group) with the decrease in the level of intake. The AME values of the diet for ducks increased from 13.43 MJ/kg (*ad libitum* group) to 13.77 MJ/kg (55% *ad libitum* group) (Table 4); however, no significant difference was detected between the two lowest levels of feeding (65% and 55% *ad libitum* groups).

The MEI of ducks at different levels of intake are shown in Table 4. The MEI of the *ad libitum* group was 1,934.25 kJ/kg of BW^0.75^/d, which decreased significantly with the decrease in level of intake as design. The RE and HP also decreased significantly with decreasing feed intake, but the decline of HP was less than that of RE. As shown in Table 5, according to the linear regression equation for RE as a function of MEI, the obtained value of ME_m_ was 625.59 kJ/kg of BW^0.75^/d, and the value of K_g_ was 0.75. Meanwhile, the linear regression equation between MEI and Log_10_HP is also presented in Table 5; the value of FHP was 549.54 kJ/kg of BW^0.75^/d, which was equivalent to the NE_m_, and the K_m_ value was calculated as 0.88.

### Energy requirements for weight gain of ducks

From the comparative slaughter experiment, the data of body composition and deposited energy of ducks at different ages are shown in Table 6. The BW and protein and energy content increased with age, whereas the carcasses moisture and fat decreased. The linear regression equation between BW and BE was established as:

$$\text{BE}=-793.02+10.41\times \text{BW }({\text{R}}^{2}=0.996,\text{root mean squared error }[\text{RMSE}]=276.822,\text{n}=15,\text{p}<0.0001).$$

From the equation, the slope of the regression equation indicated the NE_g_ was 10.41 kJ/g. According to the values of NE_g_ and K_g_, the value of ME_g_ for ducks was calculated as 13.88 kJ/g.

According to the body fat and protein deposition of ducks and the energy requirement of fat and protein deposition, the values of RE as fat (RE_f_) and RE as protein (RE_p_) at different level of intake are shown in Table 7. The values of RE_f_ and RE_p_ of the *ad libitum* group were 621.85 and 381.98 kJ/kg of BW^0.75^/d, respectively. With the decrease in level of intake, the values of RE_f_ and RE_p_ decreased significantly. The values of K_f_ and K_p_ were determined by a multiple linear regression equation of the MEI as a function of RE_f_ and RE_p_, and the values of K_f_ and K_p_ were 0.96 and 0.60, respectively, which were obtained from the following regression equation:

$$\text{MEI}=149.56+1.67\times {\text{RE}}_{\text{p}}+1.08\times {\text{RE}}_{\text{f}}\;(\text{n}=15,{\text{R}}^{2}=0.991,\;\text{RMSE}=3.623,\text{p}<0.0001).$$

## DISCUSSION

According to Latshaw and Moritz [23], broiler chickens with limited levels of feed gained less ADG and the FCR increased. Consistent with these results, we obtained reduced values of ADFI and ADG and an increase in FCR value when ducks were fed at severely limited levels. Latshaw and Bishop [24] predicted greater maintenance requirements when broiler chickens did not take enough energy daily, because the energy that was consumed had dramatic effects on the proportion of energy from feed that was used for maintenance and production. Its means the energy obtained from feed were mostly used to maintain normal physiological functions, the less energy were retention in body [25], thus, the BW increased less than normal feed intake ducks, when ducks were feed restriction.

As noted by Saleh et al [26], an increase in the availability of nutrients in diets results in changes in lipogenesis that cause changes in the composition of the carcasses. Morris [27] also found that the MEI affects the body composition of broiler chickens because animals have a tendency deposit protein rather than fat when they eat less feed. According to report of Boekholt et al [22], broiler chickens only retained protein as energy intake was restricted, but the most energy is deposited as fat when animal intake higher level energy. This might explain the increase in body protein content and reduction of carcasses moisture with the decrease in levels of feed, although the protein tissue contains more moisture than fat tissue. The value of BE decreased with the decrease in levels of feed and was related to the reduction of body fat content of ducks. Concerning gain with age, the BW, protein and energy contents increased, the carcasses moisture and fat content decreased. Results of experiment on broilers chickens, broiler pullets and quail also show that protein content increases and carcasses moisture decreases with age [28–30]. In ducks, the body fat content decreased with age, with the results in contrast to earlier studies of broiler chickens [31] and pullets [32] and quail [28,33], a discrepancy that might be due to the different ages and species, the ducks were reared for only two weeks in this experiment.

Sakomura et al [34] obtained AMEn values for broiler chickens fed 75% and 50% of *ad libitum* intake that were greater than these with *ad libitum* intake. Zancanela et al [33] found a similar performance in meat quail, which had the greatest AME_n_ value when fed 30% of *ad libitum* intake. For 21 to 42-day-old broiler chickens, the highest AME were shown at 40% of *ad libitum* intake group [25]. Consistent with these results, in the present study, the AME value of diet increased with the decrease in the level of intake, which might be due to the increase of feed digestion volume in the digestive tract caused by the decrease in the level of intake. By contrast, as the level of intake decreased in a study of growing and finishing pigs, the ME of the diet was less for pigs, and the excretion of energy in urine increased [15]. Such differences may be due to the diversity of the digestive tracts between pig and poultry, with the reduction in feed intake resulting in longer transit times through the gastrointestinal tract in pigs, therefore, an increase in the energy lost as methane.

In studies of poultry, NE_m_ values for broilers and quails have been reported. In this study, the NE requirement for maintenance was determined by a linear regression equation of FHP as a function of the ME intake of duck. Sakomura et al [31] obtained NE_m_ values of 376.52 to 499.15 kJ/kg of BW^0.75^/d for broiler chickens reared at different room temperature. As well, Liu et al [35] determined the NE_m_ for broiler chickens to be between 386 and 462 kJ/kg of BW^0.75^/d according to different methods and regression equations. Jordão Filho et al [28] determined the NE_m_ of Japanese and European quail at 18°C, 24°C, and 28°C and found values of 218.36, 207.07, and 203.43 and 240.87, 242.84, and 230.91 kJ/kg of BW^0.75^/d, respectively. In the present study, the NE_m_ values of ducks were greater than these studies about broiler chickens and quails. This may be due to the greater weight of ducks, because the NE_m_ was linearly related to metabolic BW [36]. Therefore, the temperature and the species affect the energy requirements for maintenance in birds. Besides, the rearing environment also affects the NE_m_ of birds, Jordão Filho et al [28] found that quail reared on the floor required more energy for maintenance than these reared in cages because the larger floor space allowed more locomotor activity of birds. In broiler chickens, short-term fasting significantly decrease the rectal temperature of broiler chickens [37]. With the feeding level increase, the HP increased linearly, then the heat as sensible heat loss rather than evaporative heat loss, this may be change the optimal temperature [38]. The change of optimal temperature caused by different feeding level may be led to locomotor activity. As report of Nourmohammadi et al [25], broiler chickens have reduced NE_m_ when were supplied with the pallet food and xylanase, but arabinoxylans has no effect on that [39]. Thus, there is complicated reason that influence the NE_m_ of birds.

The ME_m_ value of ducks obtained in this study was 625.59 kJ/kg of BW^0.75^/d, which is in the range of values obtained by Sakomura et al [31], who determined the ME_m_ of broiler chickens at different feeding temperature and found values of 469.03 to 660.24 kJ/kg of BW^0.75^/d. Our result was also similar to the report from Liu et al [35], which showed the ME_m_ of broiler breeding cocks were 594 and 618 kJ/kg of BW^0.75^/d by indirect calorimetry. In a study on quail, Jordão Filho et al [28] reported ME_m_ values of 443.92 and 448.11 kJ/kg of BW^0.75^/d in quail reared in cages and on the floor, respectively. However, Zancanela et al [33] found an ME_m_ value of 659 kJ/kg of BW^0.75^/d, with the discrepancy possibly due to a high breeding density. These data confirmed that birds increase HP to maintain metabolic stability when reared at temperatures above or below their thermoneutral zone. Differences among species may explain the variations in requirements for maintenance, ducks have greater BW than broilers at same age, because the ducks has higher BW than broilers.

The energy absorbed from feed is used to satisfy the requirements of maintenance for animals, and the surplus energy is deposited in body tissues as protein or lipids, which account for two-thirds of the energy requirement (NRC, 2012). Silva et al [40,41] obtained NE_g_ values for 1 to 12 and 15 to 32-day-old laying quail of 5.44 and 8.58 kJ/g, respectively. Similarly, values of NE_g_ for 0 to 14 and 14 to 35-day-old meat quail were 5.72 and 8.90 kJ/g, respectively [33]. For 3 to 8, 9 to 14, and 15 to 20-week-old broiler breeder pullets, NE_g_ values were 8.16, 7.24, and 9.37 kJ/g, respectively [42]. In the present study, the NE_g_ value for ducks was 10.41 kJ/g, which is higher than that for quail and broiler breeder pullets. This may be due to the ducks has higher growth rate than others. The increase in NE_g_ with age may be related to lower utilization when birds are close to sexual maturity. Differences in gender also affect the NE_g_ of birds [40]. In addition, ducks deposited more subcutaneous fat than chickens, as we know, and the energy requirement of fat more than that of protein [43].

In the present study, the value of ME_g_ for ducks was 13.88 kJ/g, which is higher than these for broiler breeder pullets obtained by Sakomura et al [42], who determined ME_g_ values of 3 to 8, 9 to 14, and 15 to 20-week-old broiler breeder pullets of 11.80, 10.46, and 13.56 kJ/g, respectively. Comparatively, the report of Longo et al [44] showed the ME_g_ values for 0 to 3, 4 to 6, and 7 to 8-week-old broiler chickens were 15.56, 16.65, and 17.61 kJ/g for males and 16.69, 16.44, and 29.46 kJ/g for females, respectively, which were higher than the value of ducks. The differences in species was one reason for these differences, and as noted by Zancanela et al [33], the ME_g_ is variable based on gender, lineage and species. As show as Sakomura et al [42], the ME_g_ is also related to variability in body composition, growth rate, and protein and fat deposition rates, which are affected by genetics, age, and the environment in which birds were reared.

In this study, the value of K_f_ was greater than K_p_, which was consistent with the results obtained by Zancanela et al [33], who obtained K_f_ and K_p_ values for meat quail of 0.79 and 0.32, respectively. Sakomura et al [31] found that K_p_ (0.58) was greater than K_f_ (0.55) when broiler chickens were reared at optimum ambient temperature. By contrast, values of K_f_ and K_p_ for broiler breeder pullets were 1.04 and 0.46, respectively [42]. Nieto et al [45] determined the value of K_f_ for male broilers to be between 0.65 and 1.27 and that for K_p_ to be between 0.40 and 0.58. MacLeod [46] reported that the values of K_f_ and K_p_ for female broilers were between 1.02 and 1.03 and 0.47 and 0.57, respectively. In this study, the value of K_f_ for ducks was similar to these for broilers and broiler breeder pullets, but the value of K_p_ for ducks was greater than these values. According to Sakomura et al [31], the temperature, gender, age, diet, and methods used for detection affect the values of K_f_ and K_p_. Furthermore, the K_p_ represents the ability of the animal for protein synthesis and deposition, which is relatively more species specific.

The K_m_ and K_g_ represent the efficiency of NE_m_ and NE_g_ conversion from ME_m_ and ME_g_, respectively, in this study, the values of K_m_ and K_g_ for ducks were 0.88 and 0.75, respectively. The value of K_g_ in the present study is similar to that of male growing turkeys (0.76) but higher than that of broiler chickens (0.63 to 0.69) and broiler breeder pullets (0.57 to 0.64) [31,42,47]. Working with broiler breeder pullets, Sakomura et al [42] obtained K_m_ values of 0.72 to 0.76 at different temperatures, and for broiler chickens at different temperatures, the values were 0.76 to 0.80 [31]. The greater K_m_ value obtained in the present study was expected because the ducks were less active than broiler chickens, which caused less of the MEI to be expended on daily activities. According to Jordão Filho et al [28], the K_m_ value of quail reared in cages was greater than that of quail reared on floor pens, which can be explained by the increased energy expenditure of locomotor activities on floor pens. Additionally, the dissimilarity of feed composition and fat deposition rate for growing animals contributes to such differences [45,48]. Those data further confirm that ducks are more capable of energy deposition than broilers.

In conclusion, we used the linear regression method to estimate NE_m_ and ME_m_ values of 549.54 and 625.59 kJ/kg of BW^0.75^/d, for 2 to 3-week-old Cherry Valley ducks, respectively. The requirements of NE and ME for energy deposition were 10.41 and 13.88 kJ/g, respectively, the values obtained via the comparative slaughter method. Thus, the equation to predict the NE requirements for 2 to 3-week-old Cherry Valley ducks was the following: NE = 549.54 BW^0.75^+10.41 ΔW, where ΔW is the weight gain (g).

## Figures and Tables

**Table 1:** Ingredient and nutrient levels of diet for ducks (on an air-dry basis)

Items	
Ingredient (%)	
Corn	62.9
Soybean meal	26.5
Cottonseed meal	5
Soybean oil	0.85
Unite bran	0.86
Limestone	1.16
Calcium hydrophosphate	1.46
Sodium chloride	0.3
Choline chloride	0.2
Premix1)	0.53
L-lysine	0.03
DL-methionine	0.17
Tryptophan	0.04
Total	100
Calculated nutrients	
Metabolizable energy (MJ/kg)	12.03
Crude protien	20.01
Calcium	0.90
Available phosphorus	0.40
Lysine	0.90
Methionine+systeine	0.74
Threonine	0.59
Tryptophan	0.24

1) Provided per kilogram of diet: vitamin A, 4,000 IU; vitamin D_3_, 1,200 IU; vitamin E, 18 mg; vitamin K_3_, 1.5 mg; vitamin B_1_, 0.9 mg; vitamin B_2_, 2.6 mg; vitamin B_6_, 2 mg; vitamin B_12_, 0.01 mg; pantothenic acid, 4.5 mg; nicotinic acid, 24 mg; biotin, 0.045 mg; folic acid, 0.6 mg; Cu (as copper sulfate pentahydrate), 8.0 mg; Zn (as zinc sulfate monohydrate), 90 mg; Fe (as ferrous sulfate monohydrate), 80 mg; Mn (as manganese sulfate monohydrate), 70 mg; Se (as sodium selenite), 0.3 mg; I (as potassium iodide), 0.4 mg.

**Table 2:** The effects of different feed intake on performance of 2 to 3-week-old ducks

Items	Feeding level					SEM	p-value

	*Ad libitum*	85% *ad libitum*	75% *ad libitum*	65% *ad libitum*	55% *ad libitum*		
ADFI (g)	113.47^a^	95.67^b^	83.76^c^	72.61^d^	61.51^e^	0.22	<0.001
ADG (g)	69.31^a^	58.04^b^	49.91^c^	42.70^d^	35.83^e^	0.22	<0.001
FCR	1.64^d^	1.65^d^	1.68^c^	1.70^b^	1.72^a^	0.01	<0.001

SEM, standard error of the mean; ADFI, average daily feed intake; ADG, average daily gain; FCR, feed conversion ratio.

a–e Means within a row lacking a common superscript differ (p<0.05).

**Table 3:** The effects of different feed intake on body composition of 3-week-old ducks

Items	Feeding level					SEM	p-value

	*Ad libitum*	85% *ad libitum*	75% *ad libitum*	65% *ad libitum*	55% *ad libitum*		
Water (%)	63.97^d^	64.47^c^	64.59^c^	65.18^b^	65.58^a^	0.12	<0.001
Energy (kJ/g)	9.85^a^	9.63^b^	9.48^c^	9.21^d^	9.00^e^	0.08	<0.001
Protein (%)	16.50^c^	16.75^c^	17.19^b^	17.61^a^	17.84^a^	0.21	<0.001
Fat (%)	15.64^a^	14.79^b^	13.73^c^	12.78^d^	11.89^e^	0.16	<0.001

SEM, standard error of the mean.

a–e Means within a row lacking a common superscript differ (p<0.05).

**Table 4:** The energy utilization of the diet of 2 to 3-week-old ducks

Items	Feeding level					SEM	p-value

	*Ad libitum*	85% *ad libitum*	75% *ad libitum*	65% *ad libitum*	55% *ad libitum*		
GE in excreta (MJ/kg)	14.10^a^	13.85^a^	13.93^a^	13.51^b^	13.39^b^	0.21	<0.001
AIA in excreta (%)	1.06^c^	1.06^c^	1.10^b^	1.11^a^	1.12^a^	0.004	<0.001
AIA in feed (%)	0.26	0.26	0.26	0.26	0.26	-	-
GE in feed (MJ/kg)	16.90	16.90	16.90	16.90	16.90	-	-
AM (%)	79.44^d^	79.82^c^	80.33^b^	81.11^a^	81.39^a^	0.27	<0.001
AME (MJ/kg)	13.43^d^	13.51^c^	13.60^b^	13.72^a^	13.77^a^	0.05	<0.001
MEI (kJ/kg of BW^0.75^/d)	1,934.25^a^	1,805.63^b^	1,720.48^c^	1,625.33^d^	1,498.00^e^	9.20	<0.001
RE (kJ/kg of BW^0.75^/d)	984.99^a^	892.64^b^	822.22^c^	741.27^d^	622.82^e^	5.61	<0.001
HP (kJ/kg of BW^0.75^/d)	949.26^a^	913.00^b^	898.26^c^	884.06^d^	835.19^e^	9.50	<0.001

SEM, standard error of the mean; GE, gross energy; AIA, acid-insoluble ash; AM, apparent metabolizable rate of energy; AME, apparent metabolizable energy; MEI, metabolizable energy intake; RE, retained energy; HP, heat production.

a–e Means within a row lacking a common superscript differ (p<0.05).

**Table 5:** Regression equations of RE and Log_10_HP as a function of MEI

Regression equations	R^2^	RMSE	p-value	Requirements (kJ/kg of BW^0.75^/d)		Efficiencies	
	
				NE_m_	ME_m_	K_m_	K_g_
Log_10_HP = 2.74+0.00012×MEI	0.939	0.005	<0.001	549.54	-	0.88	-
RE = 0.75 MEI − 469.19	0.993	9.605	<0.001	-	625.59	-	0.75

RE, retained energy; HP, heat production; MEI, metabolizable energy intake; RMSE, root mean square error; BW, body weight; NE_m_, net energy requirements of maintenance; ME_m_, metabolizable energy requirements for maintenance; K_m_, ME utilization for maintenance; K_g_, ME utilization for deposition energy.

**Table 6:** Body composition of ducks at different ages

Items	Age			SEM	p-value

	7 d	14 d	21 d		
Water (%)	72.55^a^	68.01^b^	63.97^c^	0.23	<0.001
Energy (kJ/g)	6.99^c^	8.41^b^	9.83^a^	0.08	<0.001
Protein (%)	14.02^c^	14.64^b^	16.50^a^	0.20	<0.001
Fat (%)	16.85^a^	15.64^b^	13.68^c^	0.31	<0.001
BW (g)	171.12^c^	572.70^b^	1,143.88^a^	6.35	<0.001
BE (kJ)	1,196.28^c^	4,815.71^b^	11,264.76^a^	55.60	<0.001
BWE (kJ/g)	6.99	8.41	9.85	9.10	<0.001

SEM, standard error of the mean; BW, mean body weight of the ducks; BE, carcasses energy content; BWE, energy content of body weight.

a–c Means within a row lacking a common superscript differ (p<0.05).

**Table 7:** The values of RE_f_ and RE_p_ of 2 to 3-week-old ducks

Item1)	Feeding level					SEM	p-value

	*Ad libitum*	85% *ad libitum*	75% *ad libitum*	65% *ad libitum*	55% *ad libitum*		
RE_f_ (kJ/kg of BW^0.75^/d)	621.85^a^	544.29^b^	468.94^c^	402.09^d^	337.92^e^	6.19	<0.001
RE_p_ (kJ/kg of BW^0.75^/d)	381.98^a^	359.81^b^	346.72^c^	332.19^d^	310.43^e^	4.94	<0.001

SEM, standard error of the mean; BW, body weight.

1) RE_f_, retained energy as fat, calculation based on energy value of fat (39.22 kJ/g); RE_p_, retained energy as protein, calculation based on energy value of protein (23.69 kJ/g).

a–e Means within a row lacking a common superscript differ (p<0.05).
